# Texture Fusion and Extreme Learning Machine for Interstitial Lung Disease Classification in HRCT Images

**DOI:** 10.3390/diagnostics16213481

**Published:** 2026-10-24

**Authors:** Maida Khalid, Maria Hanif, Jawed Mustafa, Naveed Ullah, Saqib Qamar

**Affiliations:** 1 Department of Business Analytics, Shaheed Zulfikar Ali Bhutto Institute of Science and Technology (SZABIST), Islamabad 44000, Pakistan; 2 Department of Software Engineering and Artificial Intelligence, Iqra University, Islamabad 44000, Pakistan; 3 Department of Mechanical Engineering, College of Engineering, King Faisal University, Al-Ahsa 31982, Saudi Arabia; 4 Department of Intelligent Systems, KTH Royal Institute of Technology, 10044 Stockholm, Sweden; 5 Faculty of Computing and Information Technology (FCIT), Sohar University, Sohar 311, Oman

**Keywords:** interstitial lung disease, HRCT, texture enhancement, feature fusion, PCA, extreme learning machine, SVM

## Abstract

**Background**: Accurate classification of interstitial lung disease (ILD) patterns in HRCT images remains challenging due to overlapping textures across disease types. We propose a computer-aided diagnosis pipeline for classifying five lung tissue classes from HRCT images: ground glass, micronodule, emphysema, fibrosis, and normal. **Methods**: The method applies a multi-stage texture-enhancement pre-processing step (separating coarse/fine, high/low-contrast, and directional image components) followed by extraction of nine texture, gradient and gradient-orientation descriptors (LBP, RGLBP, BDIP, bilateral, CDF, SDLGFFO, SSGSM, HOG, and gradient orientation). All nine descriptors were evaluated individually, together with one fused configuration, a combination of four of the nine (LBP, RGLBP, BDIP, and HOG), which gave the highest accuracy. Feature dimensionality is reduced using PCA guided by an eigenvalue-based estimator, and classification is performed with an extreme learning machine (ELM), with SVM (RBF kernel) used as a comparison baseline. **Results**: On a dataset of 5000 HRCT images (1000 per class; 500 train/500 test per class), the fused four-dimensional representation, classified with ELM (without kernel), achieved 92% accuracy—the best result among 46 configurations compared directly on the held-out test set and therefore a test-set-optimized figure rather than an independently validated estimate of generalization performance—outperforming individual descriptors and alternative dimensionality-reduction methods (LLE, Kernel LDA, Laplacian Eigenmaps, Nyström, Random Fourier). **Conclusions**: The proposed pipeline combines a multi-stage texture-enhancement step with multi-descriptor fusion; the enhancement step is intended to improve the separability of subtle ILD texture patterns, but its individual contribution has not been verified, as an enhanced-versus-unenhanced ablation was not performed.

## 1. Introduction

Interstitial lung disease (ILD) is an umbrella term encompassing various diseases typically centered in the lung parenchyma [1]. ILD involves similar lung areas with identical clinical, radiological, and pulmonary function findings, which complicates straightforward diagnosis. Subdivisions of ILD invite unconventional diagnosis and treatment pathways. As a leading cause of fatal conditions, ILD comprises more than two hundred chronic lung disorders. These disorders usually start with mild scarring of lung tissue and progressively worsen in form and severity if not treated in time.

**Figure 1:**
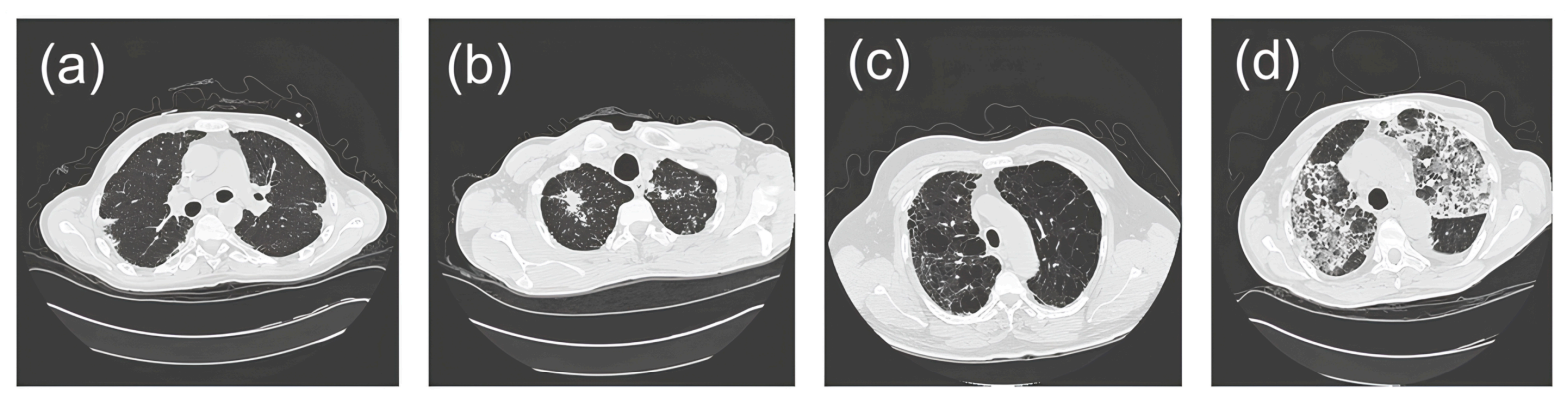
Lung disease patterns: (**a**) ground glass, a pattern of isolated diffuse opacification; (**b**) micronodule, a specific nodular pattern; (**c**) emphysema, a pattern of enlarged air-spaces; and (**d**) fibrosis, a fibrotic, scarring-like pattern.

Since ILD exhibits different pathological patterns, manual examination requires extensive diagnostic expertise. Time-consuming diagnosis includes surgical biopsy, which is hazardous and might affect the unaffected area of a lung as well. Tests such as oximetry, spirometry, diffusion testing, bronchoscopy, chest X-rays, echocardiography, and CT scans are often prescribed by physicians [2]. Computer-aided diagnosis may reduce inter-reader variation and thereby increase the accuracy of locating, detecting, characterizing, and quantifying the targeted lung region. Representative HRCT images of the four lung-disease patterns considered in this study are presented in Figure 1, illustrating their distinct radiological characteristics.

The quality of image scans also plays a vital role in the medical diagnosis of ILD. High-resolution computed tomography (HRCT) [3] is a non-invasive procedure that enables 3D anatomical visualization from any arbitrary angle. It provides visible pixel differences over dense lung pathologies, clearly indicating the qualitative and quantitative morphology of lung tissue. Due to its high diagnostic accuracy and ease of analysis, HRCT imaging is a leading modality for ILD diagnosis.

Among the available automated systems, some lack accuracy while others cannot be effectively generalized due to over-fitting. As new lung diseases emerge, there is a continuing demand for more robust and accurate diagnostic systems [4].

Texture enhancement at the pre-processing stage is rarely employed in lung disease diagnosis, yet it offers a way to make differential diagnosis more discriminating and easier. There are a number of ways to enhance image textures before supplying them to a diagnosis support system; pulmonary function tests and other clinical observations, along with patient history, have also been used as supporting evidence alongside dual-energy CT images.

Feature selection techniques employ a variety of strategies to separate abnormal from normal class clusters. Binary-pattern-based techniques and their extended variants locate class clusters using connectivity theory [5,6,7,8]. Local binary patterns in particular have been applied specifically to quantify pulmonary emphysema severity from CT texture [9]. These extended variants capture the most frequent patterns in a histogram; however, they remain restricted to common clusters. Histogram-based techniques compute gradient orientation and magnitude for each radial section of a lung disease patch [10,11]. Textural features have also been modeled using linear combinations of tissue features on low-frequency data [12]. Feature extraction of lung tissue has additionally been carried out using various statistical techniques [13], and ongoing COVID-19 research is likewise rich in feature-vector extraction using variants of these techniques [14,15,16].

Classification schemes vary considerably in their approaches to assigning class labels. Diseased lung texture has been segmented using a random forest classifier [17]. To classify different stages of systemic sclerosis, single- and multi-regression models [18] have been used. Another approach [19] exploits labeled data directly, and prior work has classified lung cancer by extracting a classical feature vector set and applying an SVM [20] to the dominant features. More broadly, computer-based analysis of lung CT has been surveyed extensively [21], and comparative benchmarks of classification algorithms applied to lung tissue categorization have shown that classifier choice interacts strongly with the underlying feature representation [22], motivating the systematic feature- and classifier-level comparison undertaken in this study.

### Related Studies

Recent work has increasingly applied machine learning and deep learning to ILD identification and characterization using data sources and tasks complementary to the HRCT texture classification addressed here. Farrand et al. [23] developed the Universal ILD Classifier, a machine-learning model trained on standardized electronic health record (EHR) data elements—diagnostic and procedure codes, laboratory results, medications, demographics, and utilization metrics-and externally validated it across multiple independent health systems, substantially outperforming rule-based, diagnostic-code-driven cohort identification; this illustrates the value of standardized, generalizable ML phenotyping for ILD but addresses cohort identification from structured EHR data rather than image-based pattern classification. Closer to the imaging domain, Fermoyle et al. [24] evaluated a deep-learning-based diagnostic support tool (SOFIA) for usual interstitial pneumonia (UIP) pattern recognition on HRCT, showing that providing algorithm output to radiologists and pulmonologists improved inter-observer agreement and prognostic accuracy across a large multinational reviewer panel—highlighting deep learning’s promise as a decision-support aid rather than a fully automated classifier. Xu et al. [25] combined CT radiomic features with clinical variables in an interpretable machine-learning (LightGBM) model to detect ILD in patients with idiopathic inflammatory myopathies, reporting strong discrimination (AUC up to 0.90) while explicitly guarding against data leakage through training-set-only feature selection, a methodological concern also central to the present study. Beyond classification, Lu et al. [26] built an integrated machine-learning risk nomogram combining clinical, dosimetric, and inflammatory predictors to forecast radiation pneumonitis in lung cancer patients with pre-existing ILD, and Raghu et al. [27] provided a state-of-the-art clinical review of pulmonary hypertension as an ILD complication, together underscoring the broader clinical significance of accurate, automated ILD characterization for downstream risk prediction and management. None of these recent studies report a directly comparable accuracy figure on the MedGIFT ILD database itself, so the 92% figure reported here cannot yet be benchmarked quantitatively against a same-dataset prior result; qualitatively, it is consistent with the strong discrimination (AUC 0.88–0.90) reported by radiomics- and deep-learning-based approaches on related ILD classification tasks above, although direct comparison awaits a shared benchmark or independent replication. Collectively, these studies reflect a shift toward standardized, externally validated, and increasingly deep-learning-based approaches to ILD-related prediction tasks; the present work differs in focusing specifically on interpretable, handcrafted texture-fusion features (expected to be computationally lighter, although runtime was not measured in this study) for direct HRCT pattern classification, which remains advantageous where large annotated training sets or deep-learning infrastructure is not readily available.

This paper proposes a new feature set built from a comprehensive combination of individual feature descriptors. The primary motivation is to facilitate a CAD system that uses a robust feature vector to localize diseased lung tissue. The proposed scheme covers five classes—ground glass, micronodule, emphysema, fibrosis, and normal—and includes a texture-enhancement pre-processing step prior to feature extraction.

To summarize, the contributions of this work are: (i) a two-stage texture-enhancement pre-processing pipeline that separates and re-combines coarse/fine, high/low-contrast, and directional image structure prior to feature extraction, whose individual contribution has not been experimentally isolated; (ii) nine texture, gradient, and gradient-orientation descriptors evaluated individually, together with a fused configuration of four of them (LBP, RGLBP, BDIP, and HOG), instead of a reliance on a single hand-picked descriptor; and (iii) an eigenvalue-guided PCA dimensionality-reduction step feeding an extreme learning machine classifier, benchmarked against SVM and against five alternative dimensionality-reduction techniques (LLE, kernel LDA, Laplacian eigenmaps, Nyström, random Fourier).

## 2. Materials and Methods

**Figure 2:**
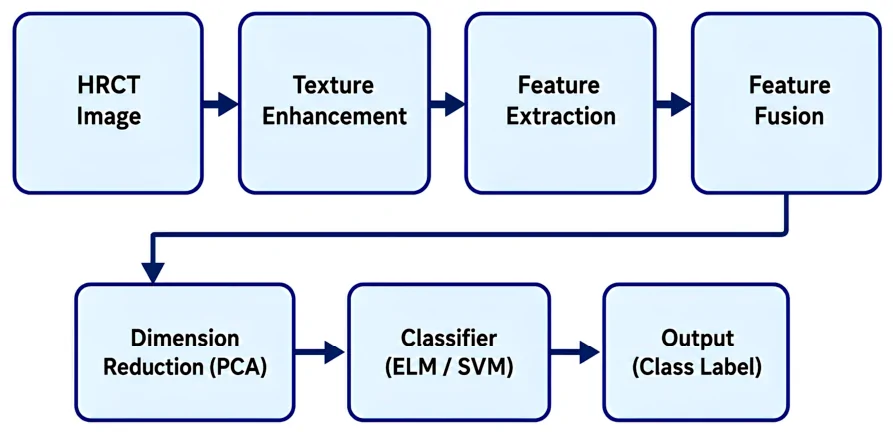
Overview of the proposed processing pipeline: HRCT image → texture enhancement → feature extraction → feature fusion → dimension reduction (PCA) → classifier (ELM/SVM) → output.

**Figure 3:**
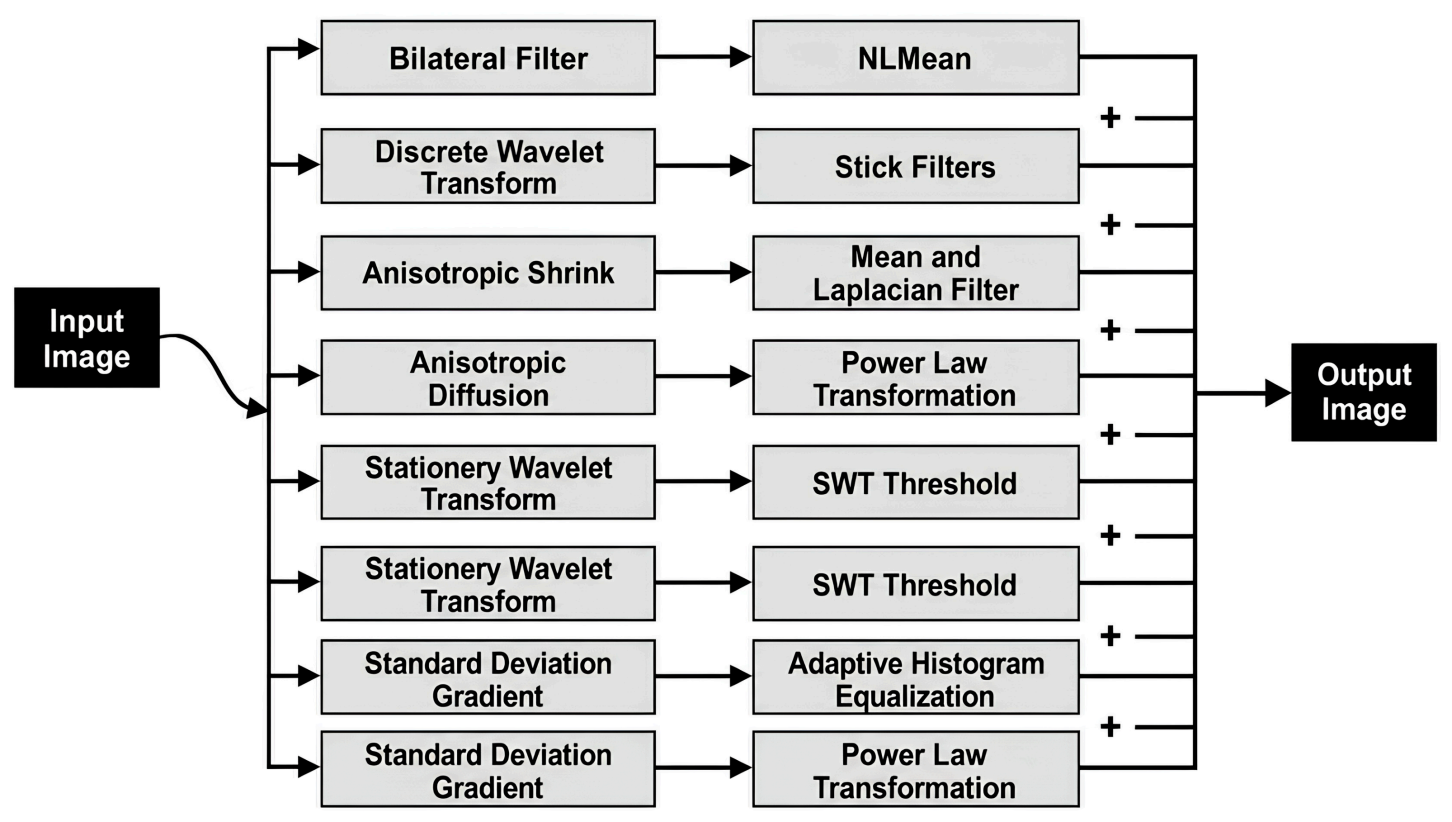
Proposed methodology showing detailed steps of the pre-processing/texture-enhancement stage. Arrows indicate the direction of data flow, and + denotes the pixel-wise summation of the enhanced components that forms the output image.

The proposed enhancement strategy is illustrated in Figure 2, with the detailed system flow shown in Figure 3. In the first stage, the input image passes through a parallel filter bank that decomposes it into coarse and fine components. In parallel, high- and low-contrast features, as well as horizontal and vertical edges, are detected while linear and non-linear details are preserved separately. In the second stage, the decomposed textures and edges are further enhanced by a second filter bank. Finally, the enhanced decomposed components are superimposed to obtain the enhanced texture image.

In brief, each stage serves a distinct purpose: the two filter banks (Section 2.1 and Section 2.2) are intended to enhance texture by sharpening coarse/fine and high/low-contrast structure so that subtle ILD patterns become more separable (this effect has not been verified experimentally); the nine descriptors (LBP, RGLBP, BDIP, bilateral, CDF, SDLGFFO, SSGSM, HOG, and gradient orientation) each capture a different texture, contrast, or gradient-orientation property of the enhanced image, and the final fused configuration retains four of them (LBP, RGLBP, BDIP, and HOG); PCA, guided by an eigenvalue-based estimator, reduces the dimensionality of the fused feature vector to mitigate the curse of dimensionality before classification; and ELM (with and without an RBF kernel) and SVM (RBF kernel) are used as the classifiers, with ELM without a kernel giving the best-performing result on the reduced fused feature set.

### 2.1. Filter Bank 1—Coarse/Fine Decomposition

(1) Bilateral Filter: Coarse components, being rich in texture, are conveniently obtained through a bilateral filter, which preserves edges by weight-averaging the gray-scale input image I ∈ Rⁱˣʲ. The pixel-wise bilateral transformation is given by

I_s,i_ = 1/W_i_ Σ_j∈S_ G_σs_(|I − j|) G_σr_(|I_i_ − I_j_|) I_j_ (1)

where I_i_ is the center-pixel value, and I_j_ is the value at the neighboring pixel j ∈ S, the set of neighboring pixels of I at position i. W_i_ is a normalization factor that converges the pixel weights to sum to 1.0. σ_s_ and σ_r_ control the amount of blurring and sharpening: as σ_s_ increases, the spatial Gaussian smooths over a wider neighborhood; as σ_r_ increases, the range Gaussian tolerates larger intensity differences between neighboring pixels.

(2) NLMEAN Filter: The coarse image obtained from the bilateral filter is passed to an NLMEAN filter, which further suppresses fine texture by computing a weighted average that enhances the coarse texture of the preceding image:

NLMEAN(I_s_) = 1/W(i) Σ_j∈ω_ u(j) f(i,j),  W(i) = Σ_j∈ω_ f(i,j) (2)

where W(i) is a normalizing factor, u(j) is the unfiltered value of the image, and ω is the set of discrete pixel values considered in the neighborhood.

(3) Discrete Wavelet Transform: Non-coarse components have less abrupt texture and hence carry the fine texture detail obtained via wavelet thresholding, preserving horizontal, vertical, and diagonal detail:

[A H V D] = dwt(I)(3)

where A, H, V, and D are the scaling, horizontal, vertical, and diagonal coefficients of the input image, respectively.

(4) Stick Filters: The fine-textured image obtained from the DWT stage is passed to a bank of stick filters, which enhance valleys and edges that are otherwise less visible, using a directional, piece-wise threshold rule with a normalization factor proportional to the stick length n. The coarse and fine enhanced images are then summed to form the final enhanced image for this branch.

### 2.2. Filter Bank 2—High/Low Contrast Decomposition

In parallel, the image I is decomposed into low- and high-contrast components.

(1) Anisotropic Shrink: Anisotropic shrink (AS) enhances high-contrast components, working in the opposite manner to anisotropic diffusion (AD):

I_s_(AS) = I_s_ + 1/|S| Σ_S∈P_ g(∇I_s_) ∇I_s_,  g(∇I_s_) = 1 − e^−(∇Is)^ (4)

The gradient g(∇I_s_) is taken so that if the local gradient is high, that area is treated as enhanced (g → 1); where the gradient is low, the region is left unchanged (g → 0), so only the high-contrast pixel regions of the input image are highlighted.

(2) Median and Laplacian Filter: The AS output is passed through a median and Laplacian filter bank to further amplify low-contrast texture:

I’_s_ = I_s_ · Medfilt(|∇^2^(C_2_)|) (5)

The Laplacian ∇^2^ is the gradient in both directions of I_s_, producing a high magnitude over discontinuous areas; the median filter then normalizes this magnitude, giving a high-contrast image I’.

(3) Anisotropic Diffusion: Low-contrast enhancement of I, denoted I_w_, is computed using anisotropic diffusion, which works similarly to AS but in the opposite direction:

I_w_(AD) = I_w_ + 1/|S| Σ_S∈P_ g(∇I_w_) ∇I_w_ (6)

### 2.3. Filter Bank 3—Horizontal/Vertical Decomposition

Image I is further decomposed into horizontal and vertical line components.

(1) Stationary Wavelet Transform (SWT): SWT with wavelet thresholding is used to specifically enhance horizontal and vertical lines of an image:

[H V] = dwt(I)(7)

(2) SWT Threshold: The image obtained from SWT is passed to a threshold operator to further enhance horizontal and vertical texture, producing two separate images:

I’(h) = SWT(I_s_(i)),  I’(v) = SWT(I_w_(i)) (8)

### 2.4. Filter Bank 4—Line-like/Non-Line-like Decomposition

Image I is subjected in parallel to line-like and non-line-like texture enhancement.

(1) Standard Deviation Gradient: A threshold value is used to separate line-like from non-line-like texture. Standard deviation gradient is used to calculate the deviation of a data point from the local mean:

SD(x,y) = Std(g(M)),  M ∈ N(9)

where SD(x,y) is the standard deviation of the gradient magnitude over a neighborhood N. Regions where SD is below a threshold δsimi are treated as non-line-like and are removed from I_s_(x,y); the converse holds for I_w_(x,y), where a higher SD marks a line-like region.

(2) Power Law Transformation: Non-line-like components are further enhanced with a piecewise power-law transformation that decompresses dark pixels while compressing bright ones:

I’_w_ = C · (I_w_)^γ^ (10)

C and γ are positive constants; γ > 1 emphasizes dark values over a wider spectrum, while γ < 1 reduces boundary and edge artifacts in non-line-like components.

(3) Adaptive Histogram Equalization: This step further enhances the already line-like-enhanced image by increasing intensity contrast over edges and boundaries, making lines and line-like edges more prominent, while decreasing contrast over the center region of the same image to remove weak intensity non-line-like components. The strong and weak enhancement filters E_s_ and E_w_ are applied to I_s_ and I_w_ respectively and summed to give the reconstructed image.

### 2.5. Reconstruction

**Figure 4:**
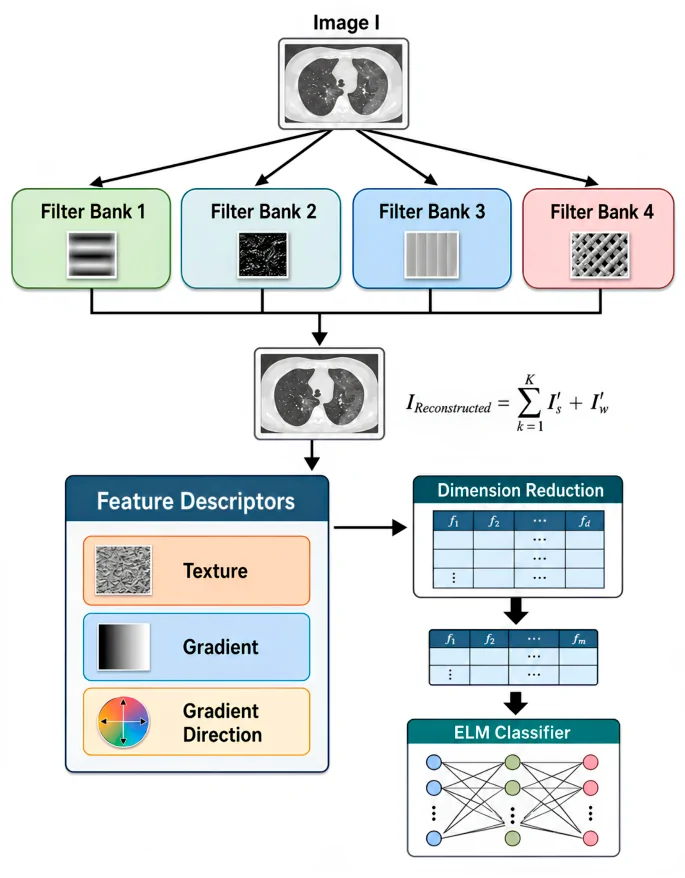
Lung-disease diagnosis system implementation and overall processing flow.

After enhancing all individual texture properties, the separately enhanced coarse/fine, high/low-contrast, horizontal/vertical, and line-like/non-line-like components are recombined:

I_Reconstructed_ = Σ_K_ (I’_s_ + I’_w_) (11)

where K indexes the number of texture components convolved with the image. All individually combined coarse/fine, high/low-contrast, and horizontal/vertical components, along with the line-like and non-line-like components, are weighted to produce the final texture-enhanced image, as summarized in Algorithm 1 and illustrated in Figure 3. The complete implementation and the sequential processing stages of the proposed lung disease diagnosis system are illustrated in Figure 4.


**Algorithm 1:** Image Texture Enhancement and Reconstruction (I)Input:  Input image I of size n × m Output: Reconstructed image I_Reconstructed
1:  Initialization 2:  Load the input image I 3:  Define enhancement filters F_s, F_w (smooth features) 4:  Step 2: Image enhancement (sharp features) 5:  for i = 1 to n (each pixel in image) do    6:    Compute sharp-enhanced image: I_s = I(x,y) · F_s    7:    Compute smooth-enhanced image: I_w = I(x,y) · F_w 8:  End 9:  Step 3: Enhancement and Reconstruction 10: For j = 1 to m (reconstruction iterations) do     11:   Apply sharp enhancement: I’_s = E_s(I_s)     12:   Apply smooth enhancement: I’_w = E_w(I_w)     13:   Compute reconstructed image:                 I_Reconstructed = Σ I’_s + Σ I’_w 14: End 15: Return I_Reconstructed


## 3. Feature Descriptors

Three main characteristics of an image are exploited for feature extraction: texture descriptors, gradient descriptors, and gradient-direction descriptors.

### 3.1. Texture Descriptors

Texture descriptors capture the rich underlying textures in lung images, conveying meaningful information. The following algorithms are used for this purpose.

(1) Local Binary Pattern (LBP): LBP is a texture descriptor centered at a pixel c, with neighboring pixels forming a circular pattern equally spaced at radius r from c. A threshold T is set for each pixel to form a binary discrete pattern: values greater than T are marked 0, and values less than T are marked 1:

LBP(P_c_) = Σ_count=0_^n−1^ U(P_count_ − P_c_) 2^count^ (12)

(2) Rotation-Invariant Gabor LBP (RGLBP): Since simple LBP alone can be less descriptive per disease class, RGLBP adds a Gabor filter “sc” and a rotation-invariance property “r”. Each image is made rotation-invariant by convolving with filter r; all rotations of a given scale are summed, and a rotation-invariant LBP histogram is computed for each scale:

RGLBP(P) = {Hist_p_ : sc = 0, …, SC−1} (13)

RGLBP(P) is constructed by concatenating the histograms of all image patches across their respective scales, producing a row feature vector rather than an image.

(3) Block Difference of Inverse Probabilities (BDIP): BDIP extracts the sketch of an image, capturing local features such as valleys and edges. An 8-bit image is divided into 8 bit planes; pixels are sampled from each plane and aggregated by usage frequency to form a binary feature vector, which is combined into the final BDIP descriptor:

I_BDIP_ = M^2^ − (Σ_Block_ I(A,B)/max_Block_ I(A,B)) (14)

(4) Bilateral Filter (as a descriptor): The bilateral filter is also used directly as a texture descriptor, preserving edges through weighted averaging of neighboring pixels using spatial and range Gaussian kernels:

I_Bilateral_ = Σ_b∈Nbr_ G_σs_(|a−b|) G_σr_(|I_a_−I_b_|) I_b_ (15)

### 3.2. Gradient Descriptors

(1) Gradient Central Differential (CDF): CDF sums the gradient taken along the x and y directions to accumulate the final edge image:

Grad_x_ = I(x−1,y) − I(x + 1,y),  Grad_y_ = I(x,y−1) − I(x,y + 1) (16)

I_CDF_ = Σ (Grad_x_ + Grad_y_) (17)

(2) Sparse Distributed Localized Gradient Fused Features of Objects (SDLGFFO): SDLGFFO is an edge-detection technique that exploits an image’s features in a hierarchical manner, respecting the spatial characteristics and anatomy through which the image is conceived. Similarly to RGLBP, it produces a row feature vector rather than an image.

### 3.3. Gradient Orientation Descriptors

(1) Spatial Stimuli Gradient Sketch Model (SSGSM): SSGSM pre-processes an image by exploiting local intensity variations under different acquisition conditions and then applies directional derivatives to compute an edge map. Bright pixels are compressed and dark pixels expanded via a logarithmic transform, after which gradients are computed in x and y, their variation is weighted exponentially, and the local stimuli map is obtained as the gradient magnitude.
(2) Histogram of Oriented Gradient (HOG): HOG captures local gradient magnitude and direction, binning them into cells and concatenating cell histograms into a final feature vector:

Grad_mag_ = √(Grad_x_^2^ + Grad_y_^2^),  Grad_θ_ = arctan(Grad_y_/Grad_x_) (18)

HOG does not produce a visual image as the other descriptors do; it produces a row feature vector directly.

## 4. Feature Dimension Estimation and Reduction

After extracting all feature-descriptor vectors, eigenvalue and correlation factors are used to estimate the dimensionality of each vector. These estimators are used alongside four dimension-reduction techniques—local linear embedding (LLE), principal component analysis (PCA), Laplacian eigenmaps, and kernel LDA—with random Fourier and Nyström methods used for comparison.

### 4.1. Local Linear Embedding (LLE)

LLE exploits the neighborhood of each data point, assuming the data lies on a smooth manifold, and preserves local linear relationships so that a lower-dimensional embedding can be mapped back to a higher dimension:

y_i_ = Σ_j∈Nindx_ w_ij_ y_j_ + e_i_ (19)

### 4.2. Principal Component Analysis (PCA)

PCA transforms correlated feature values into an uncorrelated coordinate system via orthogonal transformation so that the resulting principal components capture maximum variance:

cov = 1/n Σ (Y_t_ − mean)(Y_t_ − mean)^T^,  C_cov_ v_i_ = λ v_i_ (20)

Principal components are obtained by finding the eigenvalues that represent the maximum change in the covariance matrix; λ is the eigenvalue, and v_i_ its corresponding eigenvector. Dimension estimators (eigenvalue and correlation) are used to select the k principal components (k < d) to retain, giving the low-dimensional dataset D_low_ = Vᵀ Y_t_.

### 4.3. Laplacian Eigenmaps

Laplacian eigenmaps is a non-linear, locality-preserving algorithm that maps nearby points in the original space to nearby points in the embedded space. A weighted graph is constructed over k neighboring points, using either a Euclidean-neighbor or a nearest-neighbor rule, with edge weights computed via a heat kernel (or a simple 0/1 rule as t → ∞). Eigen decomposition of the graph Laplacian then locates the directions of maximum variation:

L v_i_ = λ D v_i_ (21)

where D is the diagonal weight matrix.

### 4.4. Kernel LDA

Kernel LDA also produces a lower-dimensional representation but does so by maximizing the between-class scatter of the n classes relative to the sum of their within-class scatter so that classes are pushed apart while each class remains compact—producing an effective, separable low-dimensional space.

### 4.5. Random Fourier and Nyström Methods (For Comparison)

Random Fourier transformation, applied via an RBF kernel, transforms an infinite feature vector into a finite one using sine/cosine projections that are easier to process in a finite space. The Nyström method instead approximates the kernel matrix directly via its top eigenvectors, producing a finite-dimensional approximation of the original feature space. Both methods are used purely as comparison baselines against LLE/PCA/Laplacian/kernel LDA in Section 7.

## 5. Classifiers

After dimension reduction [28], extreme learning machine (ELM) is used for classification, in both kernel and non-kernel forms, along with SVM with an RBF kernel for comparison.

### 5.1. ELM Without Kernel

ELM without kernel uses a randomly initialized hidden layer with an activation function (radial basis, sigmoid, or similar), which is then optimized:

f_ELM_(x) = L_hide_(x) β (22)

where L_hide_(x) maps the original feature vector into a new feature space, and β is a weight factor that is assigned randomly and then optimized.

### 5.2. ELM with Kernel

The kernel form of ELM introduces a kernel function K_kernel(x_i, x_j) = h(x_i)·h(x_j) in place of the explicit hidden-layer mapping, with β solved analytically via ridge-regression-style regularization; several kernel families (radial basis, linear, polynomial, wavelet) may be used.

### 5.3. SVM with RBF Kernel

SVM with an RBF kernel maps class labels that are not linearly separable into a higher-dimensional feature space via the RBF kernel trick, where a linear separation becomes possible:

f(y) = Σ β exp(−δ ‖y_i_ − y‖^2^) + b (23)

β controls the transformed dimension used for classification, and δ controls the spread of the RBF kernel.

Both ELM variants and SVM were evaluated on full- and reduced-dimensional feature vectors for LBP, RGLBP, BDIP, HOG, bilateral, CDF, gradient orientation, SDLGFFO, and SSGSM, individually and in combination, with the results summarized in Section 7.

## 6. Database and Evaluation Metrics

The database used in this study was the MedGIFT Interstitial Lung Disease (ILD) database, developed at the University Hospitals of Geneva (HUG) and described in Depeursinge et al. [29]. It is an open-source dataset intended for research and academic use, containing 113 DICOM series of high-resolution CT (HRCT) images with a height-by-width ratio of 512 × 512. Each image is a single-channel (grayscale) HRCT slice, i.e., 512 × 512 × 1; this matches the 262,144-element (512 × 512) raw feature vectors reported for individual descriptors in Section 7. ROIs are provided as text-file annotations and were hand-sliced by experienced radiologists. Among the 17 ILD categories available, micronodule, ground glass, fibrosis, and emphysema, together with a normal class, were selected for this research, as summarized in Table 1. Access to the database requires signing an end user agreement with the database custodian, under which authorized users may use the anonymized images and clinical data for research purposes.

Unlike previous work that relied on manually cropped image patches [30], this study used full-sized 512 × 512 HRCT images for both training and testing since patch extraction is either a tiresome manual process or an automated step that is not always accurate. To compensate for the loss of the patch-level detail that thousand-patch approaches rely on, the proposed texture-enhancement pre-processing (Section 2) was used instead.

The 5000 slices used in this study (1000 per class) were all distinct original slices drawn from the MedGIFT database; no data augmentation or oversampling was applied to inflate the class counts.

The MedGIFT database provides images organized into per-patient (per-DICOM-series) folders. The train and test sets in Table 1 were constructed at the patient level: each of the 113 folders was assigned entirely to either the training set or the test set, so no patient contributes slices to both splits. This rules out the patient-level data leakage that a naive random slice-wise split could otherwise introduce.

We do not report the exact number of patients/series contributing to each of the five classes. The MedGIFT database assigns disease-pattern labels at the ROI/slice level rather than one label per patient, so a single patient series can legitimately contribute annotated regions to more than one class (e.g., a series with both ground-glass and fibrotic regions); a simple patient-count-per-class table would therefore not sum to 113 and could be misleading without the underlying ROI-level annotation breakdown. Reconstructing this breakdown precisely requires re-parsing the original per-series annotation files, which is subject to the same dataset-access constraint noted elsewhere in this paper, rather than a simple summary we can add from memory.

**Figure 5:**
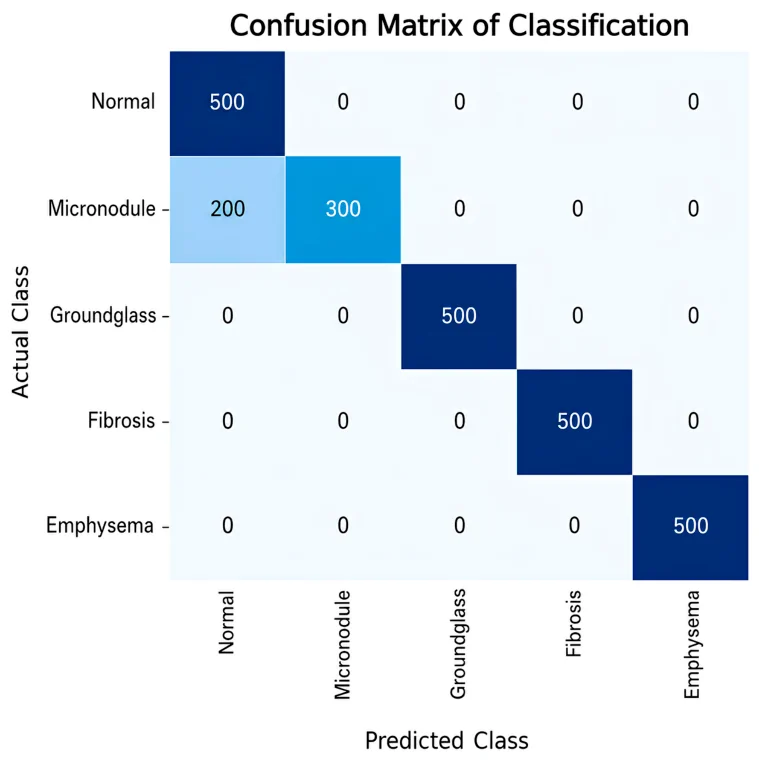
Confusion matrix for the best-performing configuration (ELM without kernel, PCA-reduced fused LBP+RGLBP+BDIP+HOG features).

Standard precision, recall, and F1-score were used to evaluate the dataset; overall accuracy is reported in Section 7, while precision, recall, and F1-score are reported for the highest-accuracy configuration (Figure 5):

Recall = TP/(TP + FN)(24)

Precision = TP/(TP + FP)(25)

F1 = 2·TP/(2·TP + FN + FP)(26)

Accuracy = (TP + TN)/(TP + TN + FP + FN)(27)

## 7. Results and Discussion

**Figure 6:**
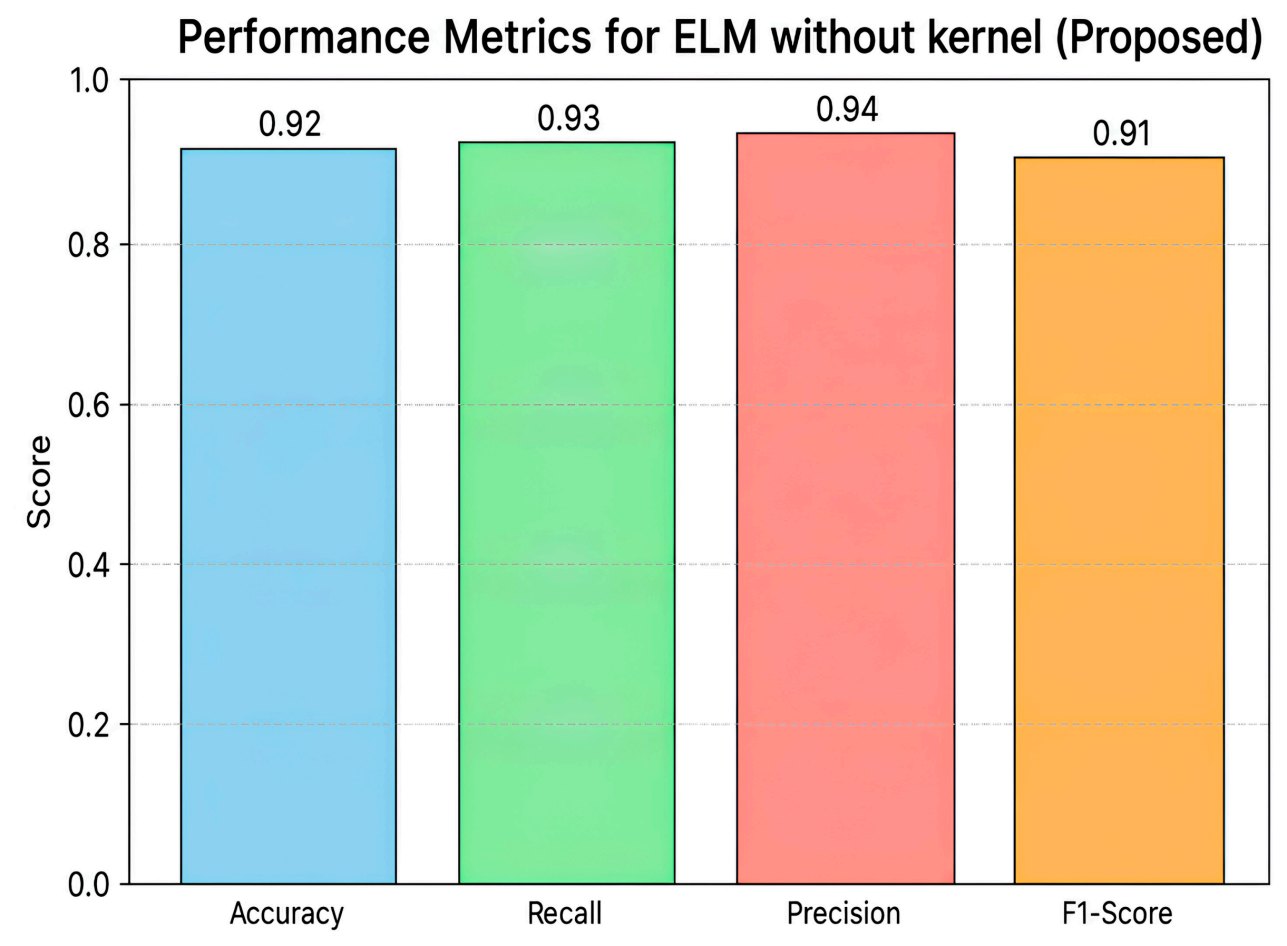
Precision, recall, and F1-score for the ELM-without-kernel configuration at its highest accuracy score.

The results of all techniques are evaluated in Table 2, and the confusion matrix for the best-performing configuration is shown in Figure 5, with the corresponding precision, recall, and F1-score shown in Figure 6. Several observations from Table 2 shaped the final configuration chosen:

(1) Using ELM with an RBF kernel on the raw (full) dimension of texture, gradient, and gradient-orientation feature vectors individually gives comparatively weak results (rows 1–9).
(2) Combining four feature descriptors—LBP, RGLBP, BDIP, and HOG—and reducing their combined dimensionality with PCA guided by an eigenvalue estimator (row 46) allows ELM without an RBF kernel to perform substantially better than any individual descriptor or alternative dimension-reduction technique, reaching 92% accuracy. Table 2 reports this single fused configuration; other descriptor combinations are not reported, and no claim is made that these four descriptors are optimal. This 92% figure represents the maximum observed accuracy across all 46 compared configurations on the held-out test set, rather than an estimate from an independent validation or nested cross-validation procedure; see Section 9 for further discussion of this limitation.
(3) The jump from individual low-dimensional descriptors (rows 38–45, topping out around 52%) to the four-descriptor, 4D fusion (row 46, 92%) is large relative to the dimensionality increase and is best understood as an interaction between descriptor complementarity and the eigenvalue-based dimension-selection procedure rather than evidence that fusion alone is responsible. LBP, RGLBP, BDIP, and HOG capture distinct, only weakly correlated image properties (local micro-texture, rotation-invariant texture, block-wise contrast, and edge-orientation histograms, respectively); when fused, their combined covariance structure differs from any individual descriptor’s, so the eigenvalue estimator selects a different, potentially more discriminative four-dimensional subspace for the fused vector than for any single descriptor compressed to the same dimensionality. This is offered as a plausible explanation for the observed gain rather than a proven mechanism; distinguishing genuine synergy from an artifact of the dimension-selection procedure would require the independent-validation and ablation experiments already identified as future work in Section 9.

In summary, the three main feature properties, i.e., texture, gradient, and gradient orientation, were exploited to obtain a set of vectors that capture the discriminating characteristics necessary for accurate diagnosis. Both reduced and non-reduced feature vectors were evaluated, and extreme learning machine was used in both kernel and non-kernel forms to compare their properties and identify the best-performing configuration.

The near-chance-level (20%) scores in Table 2 (rows 1–18) occur specifically for the ELM-RBF and SVM-RBF configurations applied to raw, full-dimensional feature vectors (up to 262,144 dimensions); every configuration using a dimensionality-reduced (4–7D) feature vector scores well above chance. This is consistent with the well-known degradation of RBF-kernel methods on very-high-dimensional, unreduced inputs (the curse of dimensionality), rather than with inconsistent optimization or evaluation protocol across methods: the same preprocessing, train–test split, and evaluation procedure were applied to every row of Table 2.

CNN-based methods were not included among the compared classifiers in this study. The handcrafted-feature pipeline evaluated here has complementary advantages over CNN-based approaches that motivate its use as a baseline: it requires no large labeled training set to learn representations from scratch, each descriptor has a directly interpretable physical meaning (texture, contrast, or gradient orientation), and inference is expected to be computationally lighter than for deep networks, although runtime was not measured in this study. A direct comparison against representative CNN architectures under the same train/test protocol would nonetheless strengthen this claim and is noted as a direction for future work.

## 8. Implementation Details

All experiments were implemented in MATLAB R2018 (The MathWorks, Inc., Natick, MA, USA). The texture-enhancement [31] stage decomposes the image via Morphological Component Analysis into coarseness, contrast, directionality, and line-likeness components using redundant SWT dictionaries (sym8 wavelet, level 3, for the coarseness, contrast, and line-likeness decompositions; Haar wavelet for the directionality decomposition), then manipulates each component: the coarse component is bilateral-filtered with spatial and range standard deviations σ_s_ = 1 and σ_r_ = 1 (window half-size 5), and the fine component is stick-filtered with stick length n = 5 and stick thickness k = 1; the high-contrast component is Laplacian/median-filtered, the low-contrast component is piecewise-power-law adjusted, the directionality components are re-weighted in a single-level sym4 SWT domain, and the line-likeness components are adjusted via a power-law transform and adaptive histogram equalization. The manipulated coarseness, contrast, and line-likeness components are recombined with fixed weights (0.05, 0.05, and 0.9, respectively) to form the enhanced image; the directionality components are computed as part of the decomposition but, in the present implementation, are not included in this final weighted recombination. The input-image normalization method and range, and, for the non-kernel ELM, the hidden-neuron count, activation function, random-initialization procedure and random seed, were not recorded and cannot be reliably reconstructed; they are therefore not reported, and the 92% result cannot be exactly reproduced from the reported settings. PCA dimensionality was not fixed a priori: the number of retained principal components was set automatically per feature configuration by an eigenvalue-based intrinsic-dimensionality estimator (‘EigValue" method). The classifier differs across the blocks of Table 2. Rows 1–9 (full-dimensional individual descriptors) and rows 28–37 (descriptors reduced with Nyström or random Fourier features) were classified using the kernel formulation of the extreme learning machine (ELM) [32], in which the random hidden-layer mapping is replaced by a kernel matrix; an RBF kernel with kernel-width parameter 5 and regularization constant C = 1024 was used for these kernel-ELM runs. Rows 10–18 (full-dimensional descriptors) and rows 19–27 (dimensionality-reduced descriptors) were classified with SVM. Rows 38–45 (individual descriptors reduced to 900 dimensions) were classified with ELM in its standard, non-kernel form. The final, best-performing configuration (Table 2, row 46: PCA-reduced, 4D fusion of LBP, RGLBP, BDIP, and HOG) was instead classified with ELM in its standard, non-kernel form (random hidden-layer mapping, regularization constant C = 102); this is the configuration reported throughout the Abstract, Table 2, and Conclusions, achieving 92% accuracy. SVM (used as a comparison baseline throughout Table 2) used an RBF kernel with γ = 0.01 and C = 1.0. All experiments were run on a standard desktop-class CPU (HP, Intel Core i5, 8th generation); no GPU acceleration was required, given the modest dimensionality of the extracted feature vectors relative to raw image data. Training and inference times were not systematically profiled, and no timing results are reported.

## 9. Clinical Limitations

While the proposed method achieves strong classification accuracy on curated HRCT slices, several clinical limitations should be acknowledged. First, ILD subtypes frequently present with overlapping or mixed radiological patterns in real patients—a single lung region [33] may exhibit both ground-glass and fibrotic characteristics simultaneously, for which this single-label classification framework does not accommodate. Extending the framework to such cases would require moving beyond single-label classification, for example through multi-label classification (allowing more than one pattern per slice), ROI- or patch-level analysis (localizing each pattern within the slice rather than assigning one label to the whole image), or explicit lesion/region segmentation prior to classification. Second, classification is performed at the slice level rather than the patient level; a definitive clinical diagnosis typically requires aggregating findings across multiple slices and correlating with clinical history, pulmonary function tests, and, where necessary, biopsy. Third, the model’s outputs have not been validated against radiologist assessments on an independent test set, which is a necessary step before any CAD system of this kind could be considered for clinical decision support. These limitations position the current work as a research prototype for feature-based lung texture discrimination [34] rather than a diagnostic tool ready for clinical deployment.

Two further limitations of the present evaluation should be noted. First, the 46 feature/classifier configurations in Table 2 were compared and selected directly on the test set, without an independent validation set or nested model-selection procedure; the reported 92% accuracy should therefore be interpreted as test-set-optimized rather than as an estimate of generalization to unseen data. Second, the contribution of the texture-enhancement pre-processing stage was not isolated experimentally since this would require re-running the pipeline on the original (unenhanced) images from the same patient-level split. Both a nested/independent-validation evaluation and a texture-enhancement ablation study are planned for future work once renewed access to the original dataset is arranged.

Three additional limitations are noted in response to further review. First, as described in Section 8, the texture-enhancement stage computes directionality components as part of its decomposition but does not currently incorporate them into the final recombined image; integrating a directionality contribution is a planned refinement. Second, the extreme learning machine results reported throughout this paper, including the 92% headline figure, come from a single run; because ELM’s hidden-layer weights (in its non-kernel form) are randomly initialized, this single-run figure does not capture run-to-run variance, and mean ± SD over multiple random initializations is identified as necessary future reporting. Third, the confusion matrix (Figure 5) shows perfect or near-perfect classification for four of five classes, with errors confined to the micronodule class; while Section 10 offers a pathology-based explanation for this asymmetry, the authors have not yet performed a formal sanity check (e.g., a class-label-shuffling test or a per-series breakdown of errors) to rule out the possibility that scanner- or series-level metadata, rather than true texture pathology, contributes to this result. Such a check is planned before the method is considered for any application beyond the present research setting.

## 10. Discussion of the Confusion Matrix

The confusion matrix (Figure 5) reveals a pronounced asymmetry in class-wise performance: while ground glass, emphysema, fibrosis, and normal were classified with near-perfect accuracy, a substantial fraction of micronodule cases were misclassified as normal. This pattern is consistent with the underlying pathology—micronodules are small, localized lesions that occupy a minority of the lung field in a given slice, so a full 512 × 512 image dominated by normal-appearing parenchyma can visually and texturally resemble a normal-class image far more than it resembles the diffuse patterns of ground glass or fibrosis. This is also consistent with the possibility that using whole-slice images rather than ROI-cropped regions dilutes the discriminative signal for spatially localized diseases such as micronodules more than for diffuse ones. This explanation is inferred from the class characteristics rather than from a direct re-analysis of the confusion-matrix values and should be validated further.

## 11. Conclusions

This work proposed a computer-aided diagnosis pipeline to classify five lung tissue classes: ground glass, micronodule, fibrosis, emphysema, and normal. A texture-enhancement pre-processing stage was proposed that decomposes and recombines coarse/fine, high/low-contrast, horizontal/vertical, and line-like/non-line-like image components prior to feature extraction. Three main feature properties—texture, gradient, and gradient orientation—were exploited to obtain a fused vector that captures the discriminating characteristics necessary for accurate diagnosis. Both reduced and non-reduced feature vectors were evaluated, and the extreme learning machine, in kernel and non-kernel forms, was used alongside SVM to identify the best-performing configuration, with the PCA-reduced fusion of LBP, RGLBP, BDIP, and HOG under ELM (without kernel) achieving the highest accuracy of 92%. This figure was obtained by selecting the best of 46 configurations evaluated directly on the test set and should accordingly be read as a test-set-optimized result; an independently validated estimate of generalization performance is identified as necessary future work.

## Figures and Tables

**Table 1:** Illustration of dataset composition.

ILD Type	#Whole Images	#Train Set	#Test Set
Micronodule	1000	500	500
Ground Glass	1000	500	500
Fibrosis	1000	500	500
Emphysema	1000	500	500
Normal	1000	500	500

**Table 2:** Classifier accuracy with full, reduced, and combined feature dimensions for ELM and SVM classifiers.

Index	Classifier	Algorithm(s)	Dimensions	DR Technique	Accuracy
1	ELM (RBF Kernel)	LBP	262,144 (Full)	—	20%
2	ELM (RBF Kernel)	RGLBP	768 (Full)	—	32%
3	ELM (RBF Kernel)	BDIP	262,144 (Full)	—	20%
4	ELM (RBF Kernel)	HOG	142,885 (Full)	—	68%
5	ELM (RBF Kernel)	Central Gradient Diff.	262,144 (Full)	—	24%
6	ELM (RBF Kernel)	Gradient Orientation	262,144 (Full)	—	20%
7	ELM (RBF Kernel)	Bilateral	262,144 (Full)	—	28%
8	ELM (RBF Kernel)	SDLGFFO	32,769 (Full)	—	20%
9	ELM (RBF Kernel)	SSGSM	262,144 (Full)	—	20%
10	SVM	LBP	262,144 (Full)	—	20%
11	SVM	RGLBP	768 (Full)	—	20%
12	SVM	BDIP	262,144 (Full)	—	20%
13	SVM	HOG	142,885 (Full)	—	72%
14	SVM	Central Gradient Diff.	262,144 (Full)	—	20%
15	SVM	Gradient Orientation	262,144 (Full)	—	20%
16	SVM	Bilateral	262,144 (Full)	—	20%
17	SVM	SDLGFFO	32,768 (Full)	—	20%
18	SVM	SSGSM	262,144 (Full)	—	20%
19	SVM (RBF Kernel)	LBP	4D	Eigenvalue-based LLE	60%
20	SVM (RBF Kernel)	RGLBP	6D	Eigenvalue with Kernel LDA	60%
21	SVM (RBF Kernel)	BDIP	4D	Correlating Dim. with LLE	60%
22	SVM (RBF Kernel)	HOG	4D	Correlating Dim. with LLE	64%
23	SVM (RBF Kernel)	Central Gradient Diff.	4D	Eigenvalue with PCA	56%
24	SVM (RBF Kernel)	Gradient Orientation	4D	Eigenvalue with PCA	28%
25	SVM (RBF Kernel)	Bilateral	4D	Eigenvalue with LLE	40%
26	SVM (RBF Kernel)	SDLGFFO	7D	Eigenvalue with Laplacian	56%
27	SVM (RBF Kernel)	SSGSM	4D	Eigenvalue-based LLE	48%
28	ELM (Kernel)	LBP	400D	Nyström	64%
29	ELM (Kernel)	RGLBP	900D	Nyström	36%
30	ELM (Kernel)	RGLBP	900D	Random Fourier	36%
31	ELM (Kernel)	Bilateral	900D	Nyström	35%
32	ELM (Kernel)	BDIP	400D	Nyström	20%
33	ELM (Kernel)	BDIP	400D	Random Fourier	20%
34	ELM (Kernel)	CDF	1600D	Nyström	40%
35	ELM (Kernel)	SDLGFFO	2500D	Nyström	60%
36	ELM (Kernel)	SSGSM	400D	Nyström	20%
37	ELM (Kernel)	HOG	2500D	Nyström	24%
38	ELM (No Kernel)	LBP	900D	Self-Selected	32%
39	ELM (No Kernel)	RGLBP	900D	Nyström	40%
40	ELM (No Kernel)	BDIP	900D	Self-Selected	48%
41	ELM (No Kernel)	CDF	900D	Self-Selected	48%
42	ELM (No Kernel)	SDLGFFO	900D	Self-Selected	32%
43	ELM (No Kernel)	SSGSM	900D	Self-Selected	52%
44	ELM (No Kernel)	HOG	900D	Self-Selected	52%
45	ELM (No Kernel)	Gradient Orientation	900D	Self-Selected	48%
46	ELM (No Kernel)	LBP, RGLBP, BDIP, HOG	4D	PCA with Eigenvalue	92%

## Data Availability

The dataset used in this study was the MedGIFT Interstitial Lung Disease (ILD) database, developed at the University Hospitals of Geneva (HUG) [29]. It is publicly available for research and academic purposes; access requires signing an end user agreement with the database custodian (HES-SO Valais), after which authorized users are granted access to the anonymized HRCT images and clinical data. Further details on the database and the access procedure are available at https://medgift.hevs.ch/wordpress/databases/ild-database/ (accessed on 19 October 2026). Additional information and processed data supporting the findings of this study are available from the corresponding author upon reasonable request.
